# Spatial analysis of prehospital emergency medical services accessibility: a comparative evaluation of the GAUSS-probability two-step floating catchment area model in Handan City

**DOI:** 10.3389/fpubh.2025.1548462

**Published:** 2025-03-28

**Authors:** Feng Tian, Saicong Lu, Zhenjie Yang, Tingting Zhao, Penghui Li, Haifang Zhang

**Affiliations:** ^1^Hebei Key Laboratory of Medical Data Science, Handan, Hebei, China; ^2^Institute of Biomedical Informatics, Hebei University of Engineering, Handan, Hebei, China; ^3^School of Medicine, Hebei University of Engineering, Handan, Hebei, China; ^4^School of Information and Electrical Engineering, Hebei University of Engineering, Handan, Hebei, China; ^5^Handan Emergency Rescue Command Center, Handan, Hebei, China

**Keywords:** spatial accessibility, prehospital emergency medical services (EMS), GAUSS-probability two-step floating catchment area (GP2SFCA) model, GIS, Handan

## Abstract

**Introduction:**

The evaluation of the accessibility of prehospital emergency medical facilities plays a vital role in the rational allocation of urban medical resources. Proper emergency medical services (EMS) planning based on accessibility evaluations can facilitate a more equitable distribution of healthcare services, thereby reducing disparities in emergency care availability across different urban areas. Prehospital EMS is often highly urgent, requiring effective intervention for patients in the shortest possible time.

**Methods:**

To meet this need, the model incorporated distance-based selection probabilities to optimize decision making by considering both geographical location and the distribution of emergency resources, making it more compatible with the practical demands of prehospital EMS. In this study, the GAUSS-Probability Two-step Floating Catchment Area (GP2SFCA) method was applied to evaluate the spatial distribution of access to prehospital EMS in Handan City and to assess accessibility differences by comparing it with various models.

**Results:**

The results demonstrated that the GP2SFCA model achieved significantly improved performance, with an average correlation coefficient of 0.7017, indicating a notably higher predictive accuracy. In contrast, other models, including the Cumulative-Opportunity Rectangular (CUMR), Kernel Density (KD), and GAUSS models, showed a much lower average correlation coefficient of 0.1542. This comparison showed that the GP2SFCAmodel achieved optimized accuracy, increasing its applicability to real world scenarios.

**Discussion:**

Our study significantly improved the evaluation and optimization of the spatial distribution of medical resources in the Handan region, offering crucial decision support for enhancing the reachability of regional medical services via its meticulous analysis of accessibility and resource allocation.

## 1 Introduction

Emergency Medical Services (EMS) provides prehospital medical care and medical transport to patients with serious illnesses or injuries that require rapid response, serving as a link between accident scenes and hospitals to ensure patients receive prompt and continuous treatment (1). Accessible and timely provision of EMS could reduce unintentional injury deaths by 60% in children and 80% in adults, which therefore is crucial to high service quality and favorable health outcomes (2, 3). However, EMSs in some areas are still mainly confronting supply demand shortages and imbalances (4). Compared with urban residents, rural residents often experienced delayed access to prehospital EMS and postponed transportation to hospital (2). With the increasing intensification of old aging, the demand for prehospital EMS has been on the increase around the world over the recent years (5).

Accessibility is often used to measure the ease at which people attain public services (2, 6, 7), and is deemed as a crucial metric for evaluating the equitable distribution of prehospital EMS. The two-step floating catchment area (2SFCA) method, initially proposed by Radke and Mu (8), but later modified and christened by Luo and Wang (9), is among the most commonly used methods for measuring spatial accessibility (6, 10). The basic 2SFCA model operates in two steps. First, the physician to population ratio was computed, which meant the number of people a physician could access within a specific driving time. Then, the ratios were summed up for every location within a specific driving time of a resident (11, 12). However, the 2SFCA method was prone to cause an overestimation of the demand for certain facility points, due to it did not differentiate distance impedance within the catchment and demand for all facility points did not vary with the number of facility points (13). Based on this, the binary decay function, a constant within the catchment and zero beyond, has become an inherent feature of the traditional 2SFCA method, which was regarded as the Cumulative-Opportunity Rectangular model (CUMR) (14). In order to overcome the limitation that the dichotomous method does not differentiate distance impedance within the catchment, the scholars introduced different distance decay functions in 2SFCA, forming a series of improved versions, such as enhanced 2SFCA (E2SFCA) (15), Gaussian 2SFCA (Ga2SFCA) (16), kernel density 2SFCA (KD2SFCA) (17), and so on. To control the competition effect between facilities when multiple facilities exist within the search radius of a demand point, Wan et al. (18) proposed a three-step floating catchment area (3SFCA) by adding a spatial impedance-based competition scheme into E2SFCA to account for a reasonable model of healthcare supply and demand. Although various modifications of the 2SFCA method have already been concerned with ameliorating the distance decay discontinuity as well as the competition effect between facilities within the search domain (14, 19), it is necessary to improve the distance decay function for the 2SFCA method in emergency scenario. It is noteworthy that multiple studies have been conducted to measure spatial accessibility to different public services, such as urban parks (20, 21), health care facilities (16, 22, 23), supermarkets (24), libraries (25), however, among these studies, little concern has been paid on the spatial accessibility assessment of prehospital EMS (5), which contributes to rational allocation and utilization of limited emergency medical resources and promotes overall population health.

This study improved the 2SFCA method by introducing distance-based selection probabilities (GP2SFCA) and used Handan, a prefecture level city in Hebei Province, as a case study to compare, analyze, and evaluate the spatial accessibility of prehospital EMS using the new model and benchmark models. Furthermore, the study examined the variations in accessibility under different parameter settings of the GP2SFCA model, providing insights into optimizing model parameters for future research. Based on this analysis, the differences in accessibility among different models were compared. These findings not only offered valuable references for prehospital EMS studies and decision making in Handan and similar regions but also explored the potential for a more rational spatial allocation of EMS resources.

## 2 Materials and methods

### 2.1 Development of the 2SFCA method

The traditional 2SFCA method (also known as the CUMR model), was carried out as previously described (14). The CUMR model assigns the same weight to all demand points within a threshold (Supplementary Figure 1). In this diagram, j1, j2, j3, and j4 represent prehospital emergency facilities, and the blue dots represent demand points, with the numbers indicating the assigned weights. This model does not consider spatial interactions between points, resulting in all demand points within the threshold receiving the same accessibility, thereby leading to uniform service access within the threshold. A kernel density (KD) function (17) or a Gaussian (GAUSS) function (16) were introduced to extend the traditional CUMR model by accounting for the distance decay of accessibility continuously in the search domain, and formed the KD2SFCA model or the GAUSS 2SFCA (G2SFCA) model, which were also carried out as previously described, respectively. The KD and GAUSS models use continuous distance decay functions to describe the influence of facilities on demand points within the catchment areas. Supplementary Figure 1 show the distance-based decay weights assigned based on the kernel density function and Gaussian function, respectively. However, conventional distance decay functions (KD and GAUSS) only apply decay within the threshold range and do not account for the distribution of emergency stations.

A Gaussian function was used continuously account for the distance decay of accessibility within a fixed catchment (7). *d*_0_ is the predetermined distance (or travel time) threshold. *d*_*ij*_ is the distance between *i* and *j*. When *d*_*ij*_ ≤ *d*_0_, the function produces a positive value that decreases as the distance increases. When *d*_*ij*_ > *d*_0_, the value of the Gaussian function is set to zero.


$$\begin{array}{l} G\left(d_{ij},d_{0}\right)=\{\begin{array}{ccc} \frac{e^{-\left(\frac{1}{2}\right)\times {\left(\frac{d_{ij}}{d_{0}}\right)}^{2}}-e^{-\left(\frac{1}{2}\right)}}{1-e^{-\left(\frac{1}{2}\right)}} & \text{, if} & d_{ij}\leq d_{0}\\ 0 & \text{, if} & d_{ij}>d_{0} \end{array} \end{array}$$

Prehospital EMS is often highly urgent, requiring effective intervention for patients in the shortest possible time. The diminishing effect of distance of the Gaussian function plays a role in the fixed catchment area decays gradually with increasing distance or time, which, however, does not consider the competition effect between facilities in the catchment area (13). In reality, the competition of multiple prehospital EMS station in the catchment area might influence the choices of patients. To address this, the study introduced the distance-based choice probability function with the denominator as the cumulative value of the distances between the demand point and all the prehospital EMS station (14, 26, 27), and developed a novel selection probability function based on the Huff mode (28) by introducing an inverse function mechanism. Thus, while introducing the Gaussian function, the distance-based choice probability function was also introduced to account for the fact that when multiple emergency stations exist within the catchment area. As shown in Supplementary Figure 1, the GP2SFCA model computes the relative selection probability of demand points for various facilities, determining which facility is most likely to be selected. Let's assume that the demand of each demand node is 10,000, and take the j3 station as an example (Supplementary Figure 2). The CUMR model assumes that all demand within the threshold range will choose the j3 station, meaning the potential demand for the j3 emergency station would be 7,000. However, this model clearly does not reflect the actual situation. The kernel density function and Gaussian function take the distance decay effect within the threshold into account. The two models (KD and GAUSS) firstly multiplies each demand node's demand by its corresponding decay weight, then sums the product of demand and decay weights for all demand nodes within the threshold range to get the potential demand for the emergency station. However, in overlapping areas, demand point k8 may have multiple choices. k8 could choose either j3 or j4. Therefore, relying solely on continuous decay models may lead to an overestimation of potential demand. The GP2SFCA model addresses this by calculating the selection probability for each facility, which can reflect the potential choice between multiple facilities, especially in overlapping service areas. The model calculates the selection probability of facilities based on the relative influence of distance, and when multiple facilities' service areas overlap, it can reasonably reflect the probability that demand point k8 will choose either j3 or j4. This approach better reflects the behavior of demand points and more closely simulates actual demand point selection behavior.

The formula for distance based selection probability was as follow:


$$\begin{array}{l} P_{ij}\left(\lambda \right)=\frac{{{\text{d}}_{ij}}^{-\lambda }}{\overset{n}{\underset{j=1}{\sum }}{{\text{d}}_{ij}}^{-\lambda }} \end{array}$$

where *P*_*ij*_(λ) is the probability that i chooses the first aid station point *j, d*_*ij*_ is the distance between the first aid station point *j* and the administrative division's (village/community) shape center *i*, and λ is the trip friction coefficient which reflects the relative importance of attractiveness in the selection process and is usually set to “2” (5, 14, 29, 30). Herein, different parameter values for λ ranging from 0.2 to 5.0 were evaluated, and found the λ range of 2.0 to 3.0 outperforming other ranges was selected for assessing the spatial accessibility of prehospital EMS (Supplementary Table 1).

The GP2SFCA calculations are categorized into two main steps:

Step 1: The formula for calculating the demand supply ratio for each prehospital emergency site was as follows:


$$\begin{array}{l} R_{j}(\lambda )=\frac{S_{j}}{\sum_{k\in \left\{d_{kj}\leq d_{0}\right\}}G\left(d_{kj},d_{0}\right)P_{kj}\text{(}\lambda ){\text{D}}_{k}} \end{array}$$

where *S*_*j*_ is the first aid station's supply capacity (we assumed that the supply capacity was the same for all stations), *G* is the GAUSS function (16), *P*_*kj*_(λ) is the probability of residents choosing the first aid station, *d*_*kj*_ is the distance between the two points, d0 is the set threshold, *D*_*k*_ is the demand within the search radius of emergency station *k* (mainly measured by the population in need of urgent emergency care), and *R*_*j*_(λ) is the supply demand ratio of the first aid station at point *j* (i.e., the service efficiency of this first aid station).

The service threshold *d*_0_ incorporated into the model defines the maximum acceptable distance to stations offering prehospital EMS in both main and non-main urban areas. Based on the guidelines issued by National Health Commission of the People's Republic of China, Health Commission of Hebei Province and other departments (31, 32), the service thresholds for main and non-main urban areas were set at 5 and 15 km, respectively. This difference accounts for the varying service coverage needs and EMS accessibility in different urban areas.

Step 2: The formula for assessing accessibility for each village/community was as follows:


$$\begin{array}{l} GP2SFCA_{i}=A_{i}(\lambda )=\sum_{j\in \left\{d_{ij}\leq d_{0}\right\}}P_{ij}(\lambda )G\left(d_{ij},d_{0}\right)R_{j}\\ \;=\sum_{j\in \left\{d_{ij}\leq d_{0}\right\}}\frac{P_{ij}(\lambda )G\left(d_{ij},d_{0}\right)S_{j}}{\sum_{k\in \left\{d_{kj}\leq d_{0}\right\}}P_{kj}(\lambda )G\left(d_{kj},d_{0}\right){\text{D}}_{k}} \end{array}$$

Where *R*_*j*_ is the service efficiency of the EMS site, *d*_*ij*_ is the distance between locations *i* and *j*, and GP2SFCA_i_ is the accessibility of prehospital EMS sites to the population at location *i*. The higher the GP2SFCA_i_ values is, the greater the accessibility of location *i* is.

### 2.2 Correlation of different distance decay functions

The demand for EMS within the search radius of every emergency station decays with distance, and service quality, as well as the location and supply of hospitals, influence residents' choice of stations. Thus, we selected the baseline models (CUMR, GAUSS and KD) as well as the newly developed GP2SFCA model to simulate the potential number of emergency cases, which was compared with the actual number of emergency cases at the emergency station. This comparison provides a reference for future researchers, facilitating further optimization of models and application methods. Pearson correlation analysis was employed to evaluate the degree of correlation between the actual number of emergency case at the emergency station and the simulated number of emergency case within the search radius of the corresponding emergency station by each distance decay function (the demand of the emergency station). Pearson's correlation coefficient and corresponding P value for each model are provided, reflecting the strength of the relationship between predicted EMS demand and actual demand. The analysis metrics also included the Mean Squared Error (MSE), Root Mean Squared Error (RMSE), Mean Absolute Error (MAE), and Mean Absolute Percentage Error (MAPE). These metrics offer a comprehensive evaluation of the model's accuracy and reliability. The correlation analysis was conducted by python 3.10.7.

### 2.3 Parameters optimization of the GP2SFCA model

Previous studies have developed a metric called Accessibility Ratio Difference (ARD) (21, 33) in order to compare the two methods in terms of accessibility levels, this approach often fails to fully account for extreme differences between regions. To address this, this study proposes the Min-Max Normalized Accessibility Difference (MMAD) metric to evaluate accessibility differences in GP2SFCA models with different λ parameters. By employing the min-max normalization method, the original linear structure of the data is preserved while normalizing the accessibility values. This standardization process harmonizes the evaluation criteria across different models, making the comparison of accessibility differences more accurate. In addition to ensuring data integrity, this method also facilitates the precise comparison of model performance across different scenarios. The formula is as follows:


$$\begin{array}{l} {\text{MMAD}}_{i}=\frac{GP2SFCA_{i}^{1}-\min(GP2SFCA_{i}^{1})}{\max(GP2SFCA_{i}^{1})-\min(GP2SFCA_{i}^{1})}\\ \;-\frac{GP2SFCA_{i}^{2}-\min(GP2SFCA_{i}^{2})}{\max(GP2SFCA_{i}^{2})-\min(GP2SFCA_{i}^{2})} \end{array}$$

${\text{GP}2\text{SFCA}}_{i}^{1}$ represents the accessibility values of the first model [with its minimum and maximum values denoted as min(${\text{GP}2\text{SFCA}}_{i}^{1}$) and max(${\text{GP}2\text{SFCA}}_{i}^{1}$), respectively], while ${\text{GP2SFCA}}_{\text{i}}^{2}$ represents the accessibility values of the second model (with min(${\text{GP}2\text{SFCA}}_{i}^{2}$) and max(${\text{GP}2\text{SFCA}}_{i}^{2}$) as its corresponding minimum and maximum values). Here, the second model uses the optimal parameter configuration derived from the analysis in Section 2.2 as the reference baseline, while the first model explores other parameter settings. MMAD is calculated to quantify the accessibility variations across these parameter configurations.

### 2.4 Study area and data description

**Figure 1:**
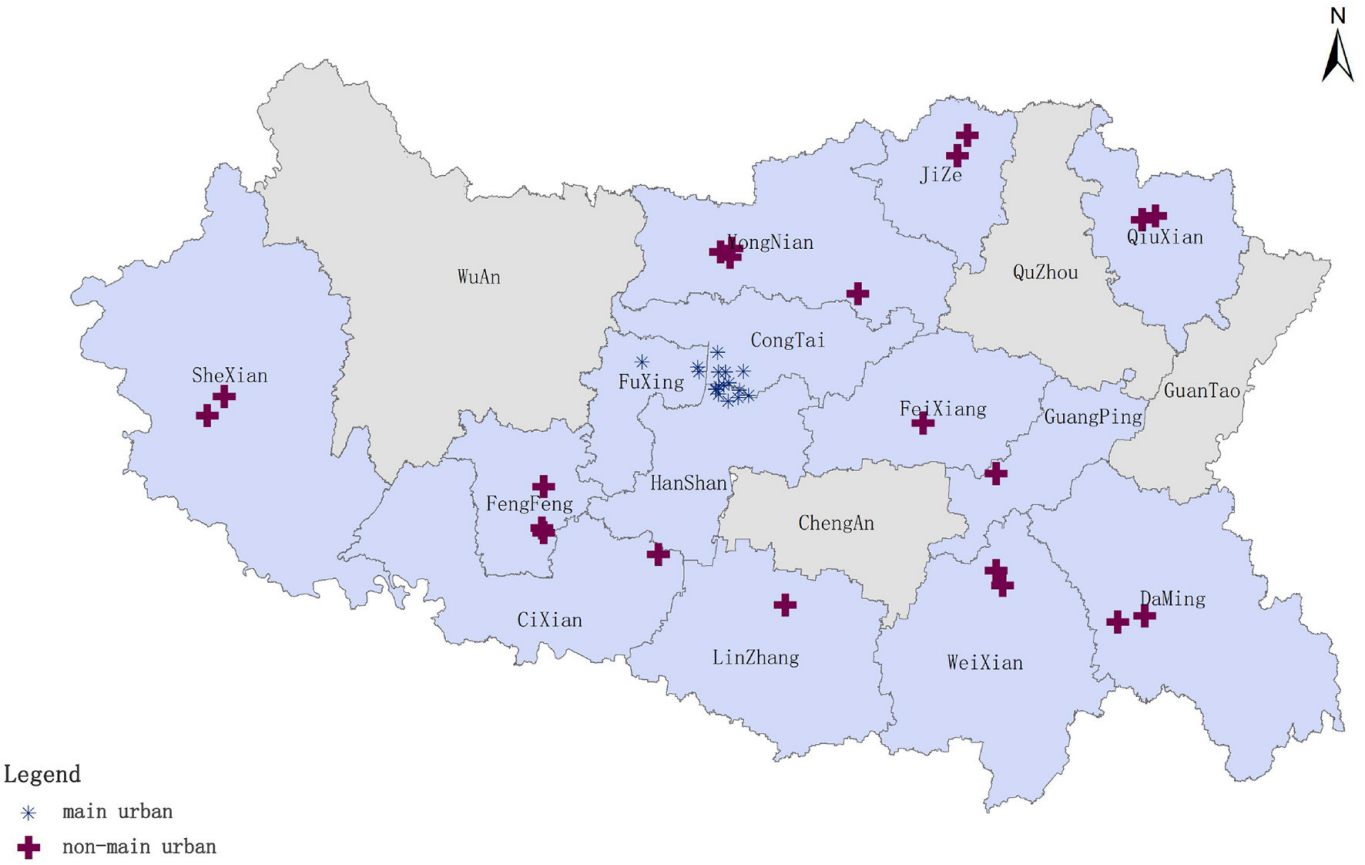
Study area and distribution of prehospital emergency sites.

To demonstrate the advantages of the improved GP2SFCA model, we applied it to measure the spatial accessibility of prehospital EMS in Handan, China. Handan, a prefecture level City under the jurisdiction of Hebei Province, is strategically positioned at the intersection of four provinces: Shanxi, Hebei, Shandong, and Henan. It is a vital node within the Beijing-Tianjin-Hebei metropolitan agglomeration and the Bohai Rim Economic Zone, located within 200 km of four provincial capitals: Shijiazhuang, Taiyuan, Jinan, and Zhengzhou. With a population of over 10 million, Handan qualifies as a mega city in China. Given Handan's substantial population, investigating the spatial accessibility of prehospital EMS is imperative. Herein, Handan was divided into two distinct zones: the main urban area comprising three major administrative districts (Congtai, Fuxing, and Hanshan) and the non-main urban area encompassing 11 county level administrative districts (Fengfeng, Feixiang, Yongnian, Linzhang, Daming, Shexian, Cixian, Qiuxian, Jize, Guanping, and Weixian). Due to data limitations, Wuan, Cheng'an, Quzhou, and Guantao, indicated by the gray areas in Figure 1, were temporarily excluded from the non-main urban study area. The final study area comprised 37 first aid station sites (16 and 21 in the main and non-main urban areas, respectively).

**Figure 2:**
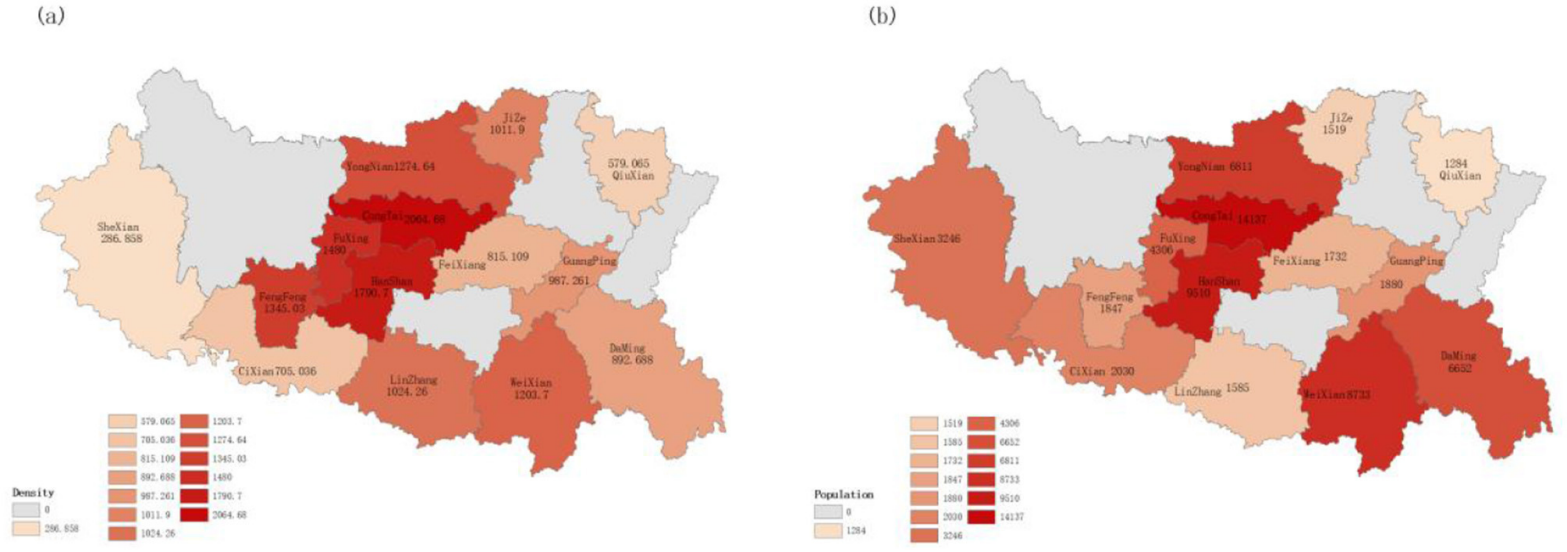
Population density and number of people in urgent need of emergency care in Handan **(A)** population density in Handan; and **(B)** number of people in urgent need of emergency care in Handan.

The prehospital emergency dispatching data for 2022, including outbound substation, delivery location, site address, and related times, were obtained from the Handan Emergency Command Center. Geocoding services were employed to convert site addresses into WGS 84 latitude and longitude coordinates, enabling the aggregation of population distribution data for urgently needed emergency care in each community or village. Herein, community and village centers were used as the demand points for prehospital emergency stations. Population data for each Handan City district and county, as well as land area statistics, were obtained from the 2021 statistical yearbook. Subsequently, the ArcGIS software was used to determine the population density distribution in Handan City and map the distribution of the population in urgent need of first aid in 2022. Handan's population was primarily concentrated in main urban areas, followed by other adjacent regions, including Yongnian, Fengfeng, and Linzhang. Conversely, Shexian, Qiuxian, and Cixian had relatively smaller populations (Figure 2A). The demand for emergency care was higher in Congtai, Hanshan, and Weixian, with Congtai recording the highest value at 14,137 people in need of emergency care (Figure 2B). Congtai's high population density justified its substantial need for emergency and first aid services. Despite having the lowest population density, Shexian registered a moderate number of emergency first aid cases. On the other hand, Fengfeng, which had the highest population density among non-main urban areas, recorded a relatively lower number of emergency first aid cases.

### 2.5 Detailed data process

**Figure 3:**
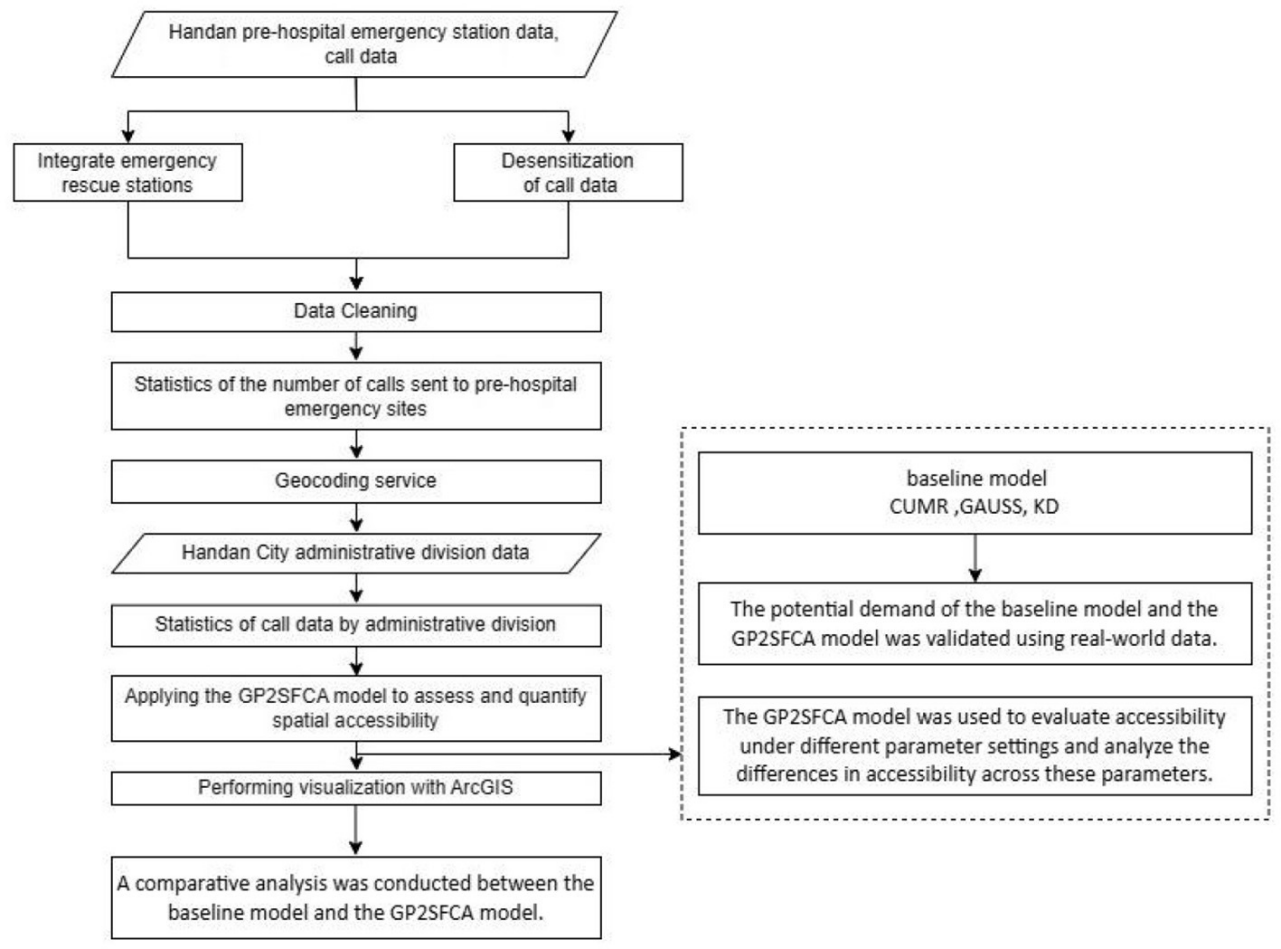
Study flow chart.

After collecting prehospital emergency station data and dispatch data from the Handan Emergency Rescue Command Center, a series of data preprocessing steps were implemented to ensure the quality of the dataset met the requirements for model validation. Figure 3 summarizes the comprehensive process that was used to analyze and interpret the spatial distribution and accessibility of EMS in the study area. First, the dataset was cleaned to remove duplicate records, thus preventing double counting during the analysis. Second, descriptions of delivery sites were standardized into a uniform format to enhance consistency across the dataset. Third, records irrelevant to the analysis, such as the empty return of ambulance, were excluded, and the number of trips to each site was also counted. Next, the EMS station addresses as well as patient address were geocoded by using python programming to obtain precise map coordinates for descriptive addresses. Then, the demands for EMS that fell within each community or village were classified and computed based on the geographical coordinates. Finally, a systematic analysis was conducted by comparing the baseline model with the newly developed GP2SFCA model, incorporating the performance of the new model under various parameter settings to explore accessibility variations. Additionally, the optimal parameter configuration of the new model was compared against the baseline model to objectively evaluate and quantify the spatial accessibility of prehospital EMS in the region. The findings were visualized using GIS technology, offering technical support for a more intuitive understanding and practical application of the results.

## 3 Results

### 3.1 Spatial clustering of prehospital emergency stations

**Figure 4:**
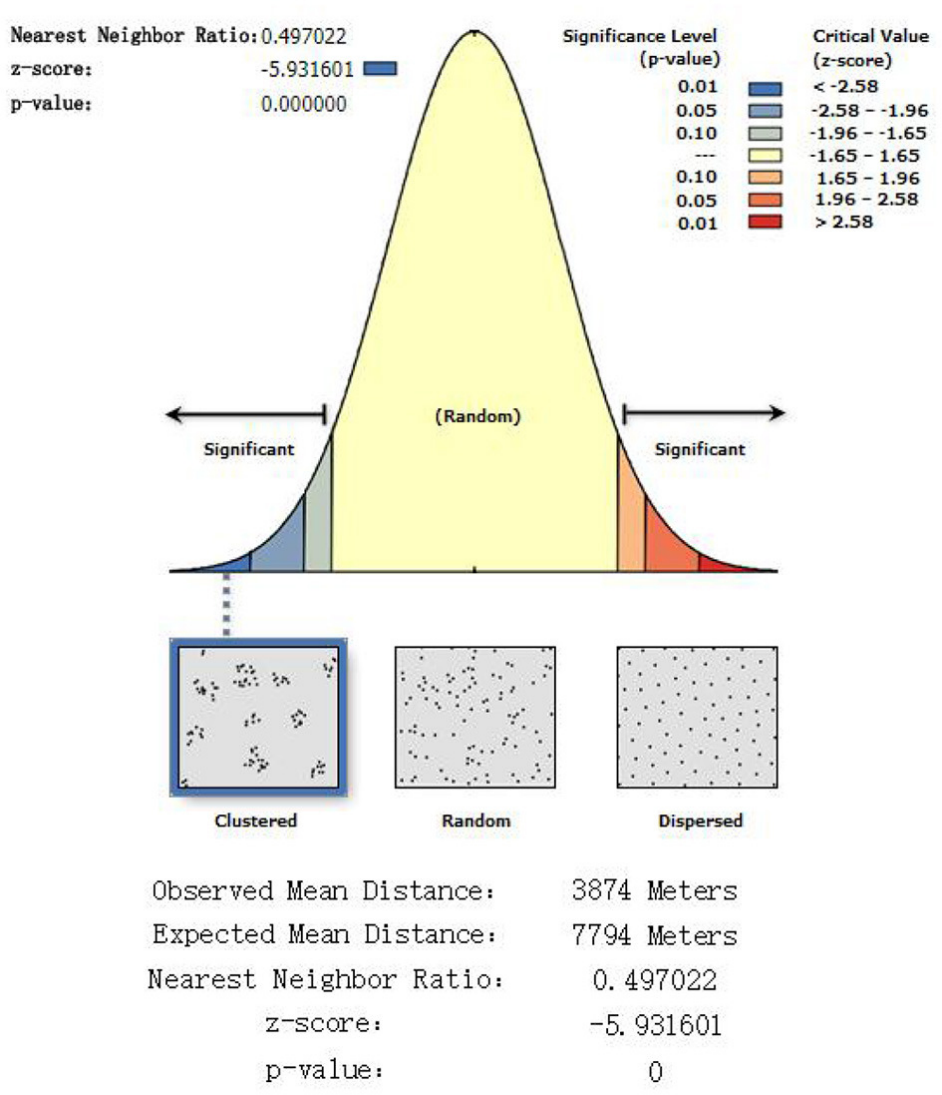
Nearest neighbor results.

As is shown in Figure 4, the spatial clustering of prehospital emergency stations in Handan was analyzed by using the “mean nearest neighbor” tool in ArcGIS. The observed mean nearest neighbor distance was ~3,874 m. Assume that the prehospital emergency stations in Handan were randomly distributed, the theoretical mean nearest neighbor distance was ~7,794 m, and the nearest neighbor ratio was 0.497 (<1, Figure 4). In addition, the *z*-value is < -2.58, and the *p*-value is < 0.001, indicating that the prehospital emergency stations in Handan are clustered and distributed.

### 3.2 Correlation analysis of different distance decay functions

To determine the degree of correlation between the actual number of emergency case at the emergency station and the simulated number of emergency case within the search radius of the corresponding emergency station by each distance decay function, the CUMR, GAUSS, KD, and GAUSS-Probability functions were selected. As shown in Table 1, the GAUSS-Probability function outperforming others, showed the strong correlation, with correlation coefficients over 0.6986 (*P* < 0.001, which means the observed correlation is extremely unlikely to occur by chance) for all λ parameters. The KD and GAUSS functions show weak correlations, with the correlation coefficients ranging from 0.2221 to 0.2293 (*P* > 0.05 for all the two functions) (Table 1). The CUMR model showed no correlation, with a coefficient of 0.0111 (*P* > 0.05; Table 1). In addition, the GAUSS-Probability function outperformed the CUMR, KD, and GAUSS functions in terms of MSE, indicating fewer squared deviations from actual values, and this trend continued in the RMSE and MAE metrics, where the GAUSS-Probability function also recorded the lowest values (Table 1). With regard to the GAUSS-Probability function, adjusting the parameter λ resulted in non-monotonic changes in Pearson's correlation coefficients, which peaked at λ = 2.4 (Table 1). Overall, the GAUSS-Probability function was more stable and reliable in the dataset, showing stronger correlations and smaller prediction errors compared to the benchmark functions (CUMR, KD, and GAUSS), and setting the parameter λ of GAUSS-Probability function to 2.4 could effectively reduce the variance in the simulation of distance decay between the actual number of emergency case and the simulated number of emergency case within the search radius of the emergency station in Handan.

**Table 1:** Correlation analysis of different distance decay functions.

**Model**	**Correlation indicator**		**Prediction error indicators**			
	**Pearson correlation coefficient**	***P*** **-value**	**MSE**	**RMSE**	**MAE**	**MAPE**
Cumulative-opportunity rectangular (CUMR)	0.0111	0.9474	294,701,390	17,167	12,582	1,923.07%
Kernel density (KD)	0.2221	0.1803	17,980,567	3,870	2,824	414.88%
Gravity-type Gaussian (GAUSS)	0.2293	0.1661	25,253,886	5,025	3,664	530.30%
**GAUSS-probability (GP)**						
GP2.0	0.6999	1.01e-06	2,185,230	1,478	995	75.80%
GP2.2	0.7026	8.76e-07	2,090,059	1,446	968	76.67%
GP2.4	0.7038	8.23e-07	2,013,611	1,419	944	77.16%
GP2.6	0.7027	8.73e-07	1,951,016	1,397	923	77.78%
GP2.8	0.7022	8.96e-07	1,898,607	1,378	906	78.25%
GP3.0	0.6987	1.07e-06	1,860,807	1,364	891	78.71%
Average	0.7017	9.24e-07	1,999,888	1,414	938	77.40%

### 3.3 Analysis of spatial accessibility across different λ parameters

**Figure 5:**
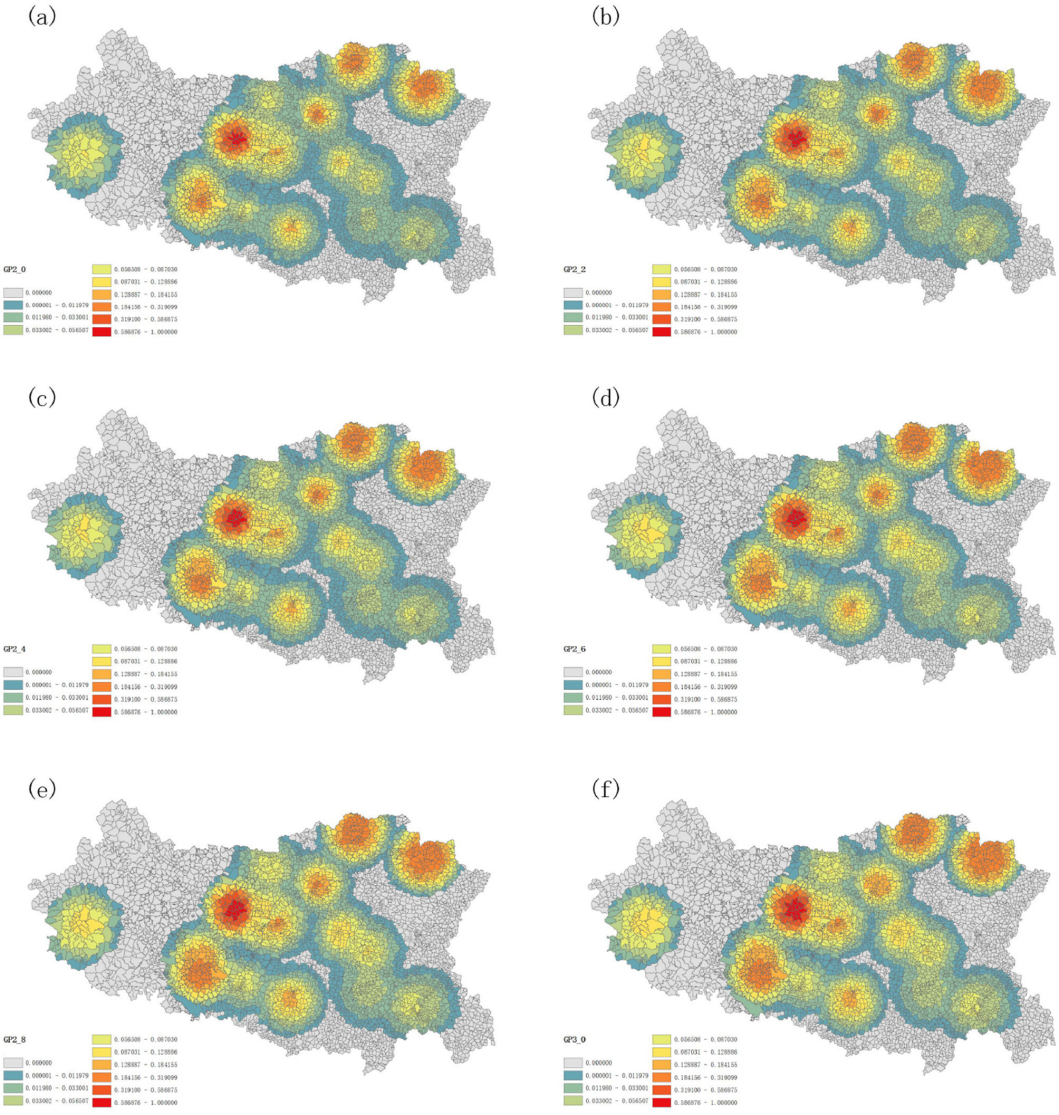
Spatial accessibility distribution with λ set at 2.0 **(A)**, 2.2 **(B)**, 2.4 **(C)**, 2.6 **(D)**, 2.8 **(E)**, and 3.0 **(F)**.

Herein, the GP2SFCA model was used to explore the differences in spatial accessibility across various parameters. The parameters analyzed included λ values of 2.0, 2.2, 2.4, 2.6, 2.8, and 3.0. As shown in Figure 5, those regions that have high spatial accessibility ratios were shown in red, and with the decrease of accessibility, the colors of regions dyed in the image gradually faded. On the whole, the same accessibility patterns of prehospital EMS in Handan were revealed approximately across different λ parameters. However, as the λ parameter increasing, areas of poor accessibility constantly shrunk on the one hand, on the other hand, areas that have high accessibility kept expanding, which meant the low λ parameter was prone to underestimate the spatial accessibility while the high λ parameter was likely to overestimate the spatial accessibility of prehospital EMS in Handan, and medium sized λ parameter should selected to reduce the bias in the estimation of spatial accessibility to prehospital EMS in Handan (Figure 5).

**Figure 6:**
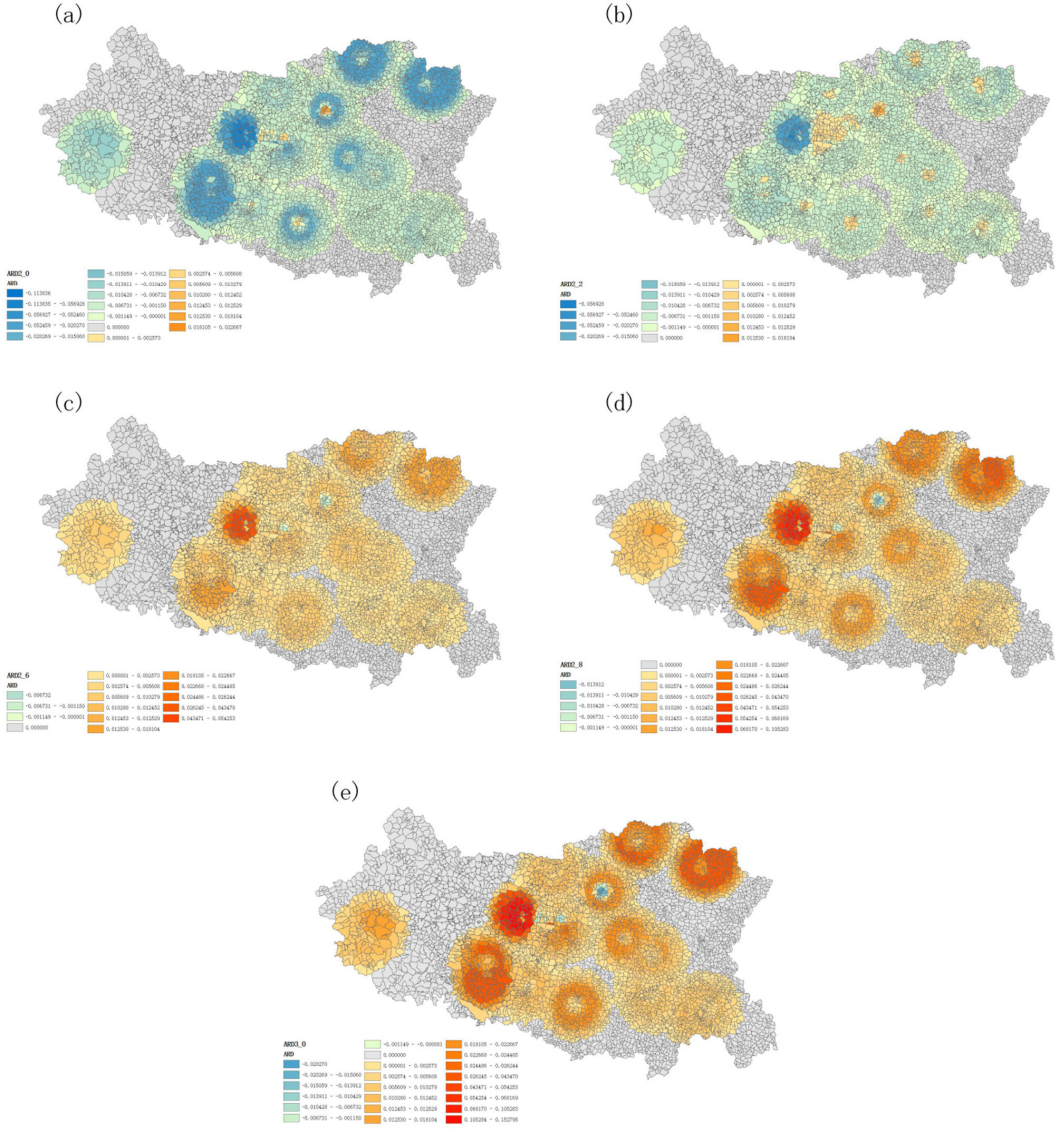
Accessibility discrepancies across different λ parameters of the GP2SFCA model. **(A)** Accessibility difference at λ = 2.0 compared to λ = 2.4; **(B)** Accessibility difference at λ = 2.2 compared to λ = 2.4; **(C)** Accessibility difference at λ = 2.6 compared to λ = 2.4; **(D)** Accessibility difference at λ = 2.8 compared to λ = 2.4; **(E)** Accessibility difference at λ = 3.0 compared to λ = 2.4.

To vividly depict the bias in the estimation of spatial accessibility to prehospital EMS in Handan, the MMAD, a min-max normalization method was employed to compare the accessibility discrepancies across different λ parameters. The standard λ parameter was set to 2.4, which was used to compute the referenced spatial accessibility by using the GP2SFCA model. For the two low λ parameters (2.0 or 2.2), the calculated spatial accessibilities by using the GP2SFCA mode roughly showed negative accessibility discrepancies (the MMAD values <0) compared with that of the referenced λ parameter (Figures 6A, B). On the contrary, the spatial accessibilities to EMS in Handan calculated by the high λ parameters (2.6, 2.8, or 3.0) harbored positive discrepancies (the majority of MMAD values >0) compared with that of the referenced λ parameter (Figures 6C–E). These results indicated that setting the λ parameter to 2.4 could effectively reduce the bias in the estimation of spatial accessibility to prehospital EMS in Handan, which should be used as a recommended index of the GP2SFCA model in the further research.

### 3.4 Accessibility analysis of prehospital EMS in Handan

**Figure 7:**
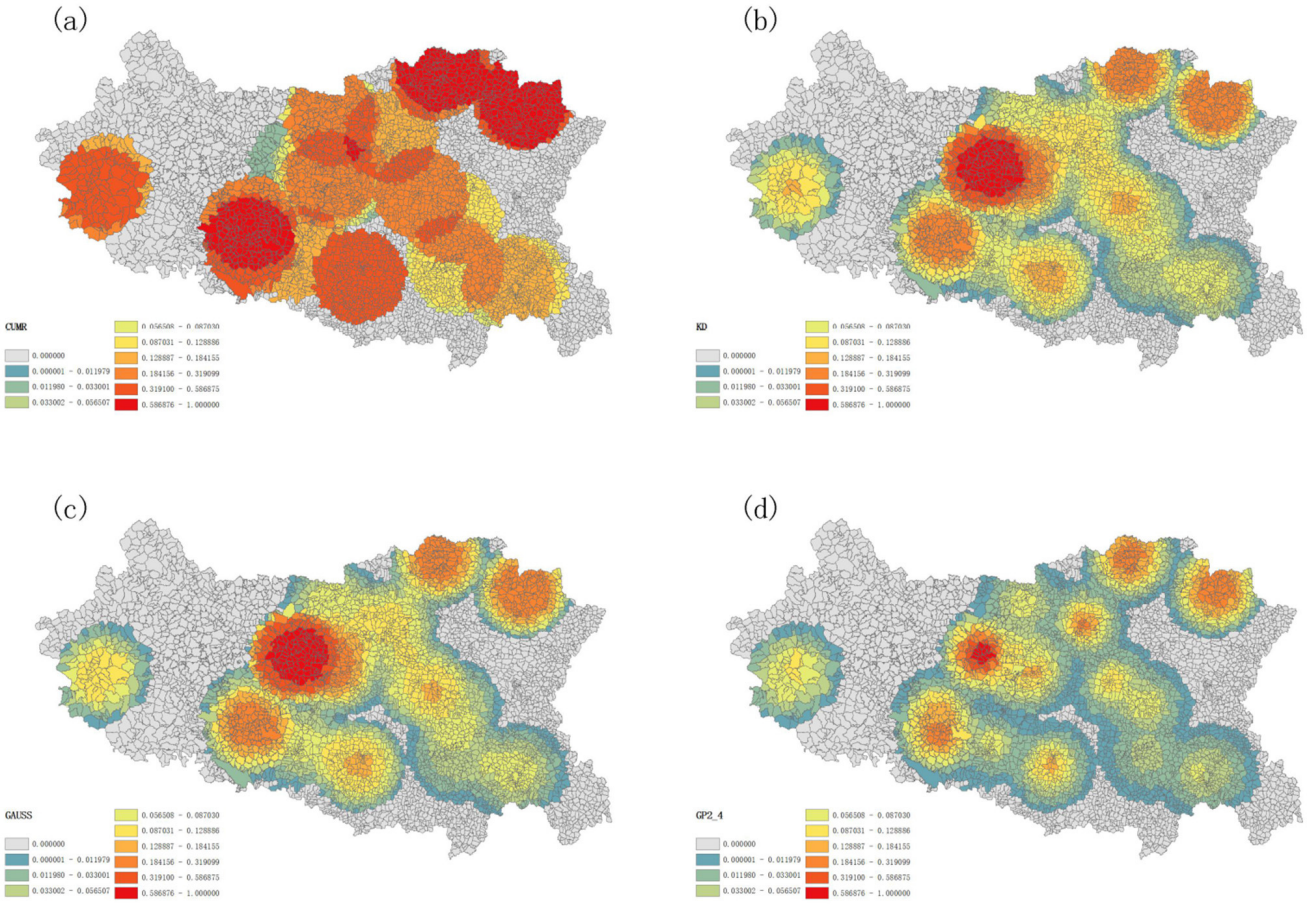
Accessibility of different models for prehospital emergency care **(A)** assessing accessibility for the CUMR, **(B)** assessing accessibility using KD as a distance attenuation function, **(C)** assessing accessibility using GAUSS as a distance attenuation function **(D)** assessing accessibility for the GP2SFCA model.

To accurately describe the accessibility of prehospital EMS in Handan, the optimal GP2SFCA model was used with parameter λ = 2.4. Meanwhile, for comparison, the CUMR model, the KD2SFCA model and the G2SFCA model were also applied for the accessibility analysis of prehospital EMS in Handan. The CUMR model, the traditional 2SFCA method, did not consider the effect of distance, resulting in uniform accessibility within the same region (Figure 7A). By incorporating a distance decay function, the KD2SFCA model and the G2SFCA model showed that accessibility varies within the same region due to distance factors (Figures 7B, C). As is shown in Figure 7D, the GP2SFCA model revealed more details about reachability relative to baseline models, to be specific, accessibility to prehospital emergency resources varied significantly across Handan, with an uneven distribution of spatial accessibility. In general, the accessibility values of EMS resources in the southwest and southeast of the city are generally low, while the central city and the north of the city are areas with relatively high accessibility values (Figure 7D). The villages with the highest spatial accessibility to prehospital EMS were Zhangyanyu, East Changshe, Caihe, Niujiaohe, Suzhuang, Hu, and West Changshe, which located in Fuxing district, one of the central urban areas (Figure 7D). Among the 14 counties (districts), Fuxing showed the highest accessibility of prehospital EMS, the following were Qiuxian, Fengfeng, Jize, Congtai and Hanshan, Weixian, Daming and Shexian demonstrated lower accessibility (Figure 7D). Taken together, the overall accessibility of prehospital EMS to residential areas varies significantly, with accessibility decreasing from the center to the marginal regions.

## 4 Discussion

We have proposed an improved 2SFCA method (GP2SFCA model) by integrating the G2SFCA model with a probabilistic function to determine the probability of choosing the first aid station in each community or village based on distance. The GP2SFCA model was used to assess the spatial accessibility of prehospital EMS in 14 counties (districts), and to compare their geospatial differences in Handan.

As far as we know, several modifications of the 2SFCA method have already been reported to improve the distance decay discontinuity within the search domain (10, 16, 17, 34). Dai (16) emphasized the importance a Gaussian function in the 2SFCA method with to estimate the health care access, while Polzin et al. (34) and Dai and Wang (17) highlighted the critical role of the Kernel Density function in the 2SFCA model to measure the spatial access to health care and to food stores in southwest Mississippi, respectively. However, multiple factors, such as the location and supply of public services, influence residents' choice of stations, it will be essential to consider the most probable emergency stations residents will choose. In the study, we determine firstly the degree of correlation between the actual number of emergency case at the emergency station and the simulated number of emergency case within the search radius of the corresponding emergency station by the CUMR, KD, GAUSS and GAUSS-Probability functions, and found that the GAUSS-Probability function outperformed the CUMR, KD, and GAUSS functions, showed the strong correlation between the actual number of emergency case and the simulated number of emergency case within the search radius and fewer squared deviations from actual values, illustrating the immense potential of the GAUSS-Probability function in the application of the 2SFCA model to describe the accessibility of prehospital EMS.

In previous accessibility analysis methods based purely on distance friction, the friction parameter is typically set to 2 (5, 14, 29, 30), which is a common practice. A sensitivity analysis on λ, setting λ as 1 or 2 respectively was conducted, and the results showed that 2 is better for measuring the accessibility of healthcare facilities (5, 35). However, it is different for emergency station compared to other health care facilities. Therefore, in preliminary experiment, we tested a set of coefficients ranging from 0.2 to 5.0 with an increment of 0.2, exploring the impact of different parameters on model accessibility. It was found that the λ range of 2.0–3.0 outperforming other ranges <2 or >3, showed a stronger correlation between the actual number of emergency case at the emergency station and the simulated number of emergency case within the search radius of the corresponding emergency station, and could more accurately balance the influence of distance on the choice of emergency services by patients and reflect the spatial distribution of emergency services in different regions, especially in urban-rural fringe areas and remote regions, ensuring a reasonable and optimized allocation of emergency resources. This provides more insight into selecting the optimal parameter. By incorporating a distance-based selection probability function into the model, it is more accurately accounted for the influence of different facilities on accessibility calculations. This enhancement enables the model to reflect both the distance decay effect and the probability of selecting facilities, thus improving the realism and accuracy of the results. In the study, we selected different parameter values of 2.0–3.0 for λ, and used the GP2SFCA model to explore the differences in spatial accessibility across various parameters. We demonstrated that the low λ parameter was prone to underestimate the spatial accessibility while the high λ parameter was likely to overestimate the spatial accessibility of prehospital EMS in Handan, and setting the λ parameter to 2.4 has shown promising results in reducing bias in the spatial accessibility estimation of prehospital EMS in Handan. Preliminary findings suggest that this parameter configuration could be a useful reference for further studies on similar models. However, broader validation is needed to confirm its applicability and ensure its reliability in diverse scenarios.

We integrated the CUMR model (the traditional 2SFCA method) with different distance decay function to model accessibility of prehospital EMS and lead to varied implications for spatial planning and analysis. As expected, our optimal GP2SFCA model with parameter λ = 2.4, outperformed the CUMR, KD2SFCA, and G2SFCA models. The CUMR model evaluates accessibility by purely considering the supply demand relationship without accounting for the distance between supply and demand points or the attractiveness of facilities (14). Because it ignores geographic factors, the CUMR model yielded a uniform accessibility rating within the same region, and overlooked the distance factors induced nuanced differences in accessibility of prehospital EMS in Handan. Both the KD2SFCA and G2SFCA models incorporate distance decay functions. The KD model used a flat or slightly modulated curve based on the kernel chosen, while the GAUSS model applies a Gaussian curve to simulate how accessibility diminishes with distance (14, 36). Here, the KD2SFCA model and the G2SFCA model showed that accessibility varies within the same region due to distance factors, highlighting the impact of proximity on access to emergency services. The GP2SFCA model considers proximity to estimate the probability of selecting a specific emergency station in urgent situations, thereby improving accessibility evaluation. This enhancement provides more detailed insights into accessibility compared to the baseline model and aligns more closely with real world scenarios. Moreover, by incorporating such practical considerations, the GP2SFCA model provides a more realistic and applicable framework for planning and optimizing healthcare services. The GP2SFCA model adopted in this paper has the following advantages: (1) The GP2SFCA model provides higher accuracy in predicting how likely residents are to choose specific medical facilities based on distance. This alignment with real world scenarios better captures the complex scenarios of decision making in emergency medical situations. (2) The model's enhancements were validated using actual data from Handan. It was observed that the improved GP2SFCA model outperformed traditional models in key performance indicators such as accuracy and reliability of resource accessibility assessments. (3) Analyzing the spatial distribution of accessibility differences for various parameters helps the identification of areas that are particularly sensitive to changes in model parameters. This analysis can reveal regions where accessibility is either significantly better or worse, guiding targeted improvements. (4) This study analyzes multiple models and compares their differences in accessibility. Utilizing ArcGIS for spatial analysis and visualization provides a detailed view of how medical services are geographically distributed. (5) The proposed model can provide more detailed insights into spatial accessibility to different public services, such as healthcare, compared with the existing models, and serve as a tool to guide healthcare system design and policy formulation. For example, policymakers can identify regions with lower accessibility to prehospital EMS and implement targeted interventions by utilizing model. This approach aligns with frameworks that emphasize the importance of data driven decision-making in healthcare policy (37).

The evaluation of EMS demand was the first step in the implementation of the GP2SFCA model. The EMS demand was mainly measured by the population in need of urgent emergency care. In determining need, the distribution of persons within the area of interest emerges as an important factor (38, 39), thus, the distribution of total population as well as the population needing EMS in Handan were mapped using GIS technology. In addition, demographic factors (such as age, gender, or health status) were important in determining the EMS needs for a specific geographic unit. Age was an important factor for EMS demand, especially among the older adult. Older adults generally face higher health risks, such as cardiovascular and respiratory diseases, which leads to a significantly higher demand for EMS compared to other age groups (11). Impact of gender on EMS demand was that women had higher EMS needs for emergency obstetric and newborn care, while men tended to emergencies involving injuries, poisoning, and external causes. Health status also had a significant impact on EMS needs. Specifically, poor health conditions could increase the demand for EMS, as some chronic and acute illnesses might lead to sudden situations that required urgent medical help. Ignoring the demographic factors might lead to the inaccurate prediction for EMS demand of specific populations, which should be taken seriously in the Future research. In this study, the EMS data in Handan was also employed in the implementation of the GP2SFCA model due to the fact that patient-centered evaluation of healthcare services was crucial to establish evaluation tools which could be used to improve quality of care and patient experience (37).

Our study elucidated the spatial accessibility of prehospital EMS in Handan by using the GP2SFCA model. It was found that accessibility to prehospital emergency resources varied significantly across Handan, with an uneven distribution of spatial accessibility. For the main urban area, the accessibility of prehospital EMS was in the order as Fuxing district > Congtai district > Hanshan district. Despite a lower density of first aid stations than Congtai district and Hanshan district, Fuxing district showed higher accessibility, owing to its lower population density and strategic location close to the boundary with Congtai, where it benefited from Congtai's resources, resulting in more resources per capita within the service radius at the community and village levels. Furthermore, the Qiuxian and Jize areas showed higher overall accessibility values, potentially due to a lower emergency care demand. On the other hand, Weixian demonstrated lower accessibility due to its higher population density and greater emergency care needs. Daming also showed lower accessibility due to its high emergency care needs. Overall, there was a notable pattern of higher accessibility in central areas and lower accessibility in the marginal regions, implying a service provision gradient that diminished with distance from the location of the central emergency resources. In the central areas of Handan City's administrative divisions, although the peripheral regions may access some resources from neighboring areas, they still exhibit relatively low accessibility. In the boundary areas of the outermost administrative divisions of Handan City, residents face challenges in accessing prehospital emergency medical resources in a timely manner or encounter significant difficulties in doing so due to factors such as distance. Future efforts can focus on selecting appropriate emergency station locations in these underserved areas to improve resource distribution and service coverage.

### 4.1 Future applicability and limitations

There are some limitations for this study. First, in current literature, medical facility capacity is measured using quantifiable metrics like beds, doctors, and nurses, which may not suit pre-hospital emergency services. New methods should be developed to assess the supply capacity of pre-hospital emergency facilities, tailored to their unique operations and critical services. Second, we adopted distance-based metrics to evaluate the accessibility. The service thresholds for main and non-main urban areas were set at 5 and 15 km (31, 32), however, such fixed distance thresholds overlooked the actual road network, might result in potential discrepancies between the calculated travel distances and the real distances. Actually, travel time is likely more critical than distance in prehospital EMS, the time delays of ambulances in prehospital EMS were no obvious between the morning or evening rush hours and other parts of the day, due to the fact that ambulances in China have priority access when performing emergency tasks. Future research should consider utilizing the Driving Searching API of online map that provide more accurate travel time estimation from demand locations to hierarchical facilities, thereby providing a more precise assessment of EMS spatial accessibility. Third, attractiveness factors (such as hospital grade, areas of expertise, hospital capability, etc.) and patient characteristics (such as age, gender, category of disease, etc.) were not considered. The attractiveness factors will have an impact on the choice of prehospital EMS. Ignoring the patient characteristics might lead to the inaccurate prediction for EMS demand of specific populations, which should be taken seriously in the future research. In the future, a comprehensive index should be developed based on the indicators that reflect the above factors. Building on this, genetic algorithms and clustering algorithms should be used to develop location selection models for optimized site selection.

## 5 Conclusions

This paper presented the GP2SFCA models, an improved 2SFCA method, for analyzing and evaluating the spatial accessibility to prehospital EMS in Handan by integrating the 2SFCA model with the GAUSS-Probability function, which was more stable and reliable in the dataset, showing stronger correlations and smaller prediction errors compared to the benchmark functions. Utilizing the GP2SFCA model, we examined the spatial accessibility of Handan's prehospital EMS. The centralized distribution of emergency stations could reduce accessibility in surrounding areas. The findings of this study not only extend the current understanding of spatial accessibility of prehospital emergency medical facilities in Handan City but also offer empirical support for the scientific and effective planning and allocation of medical resources. By highlighting areas with inadequate EMS accessibility and identifying the efficacy of different modeling approaches, our study will serve as a guide for policymakers and planners in making informed decisions that can significantly improve emergency medical response scenarios and service provision, particularly in underserved areas.

## Data Availability

The original contributions presented in this study are included in this article. Further inquiries regarding additional data can be directed to the corresponding author. Requests to access these datasets should be directed to lipenghui@hebeu.edu.cn.
