# Supplementary material for: Spatial analysis of prehospital emergency medical services accessibility: a comparative evaluation of the GAUSS-probability two-step floating catchment area model in Handan City
Source: Front Public Health. 2025 Mar 28;13:1548462. doi: 10.3389/fpubh.2025.1548462 (PMC11986854; doi:10.3389/fpubh.2025.1548462)
Supplement: Supplementary file 1 [file Data_Sheet_1.docx]

Supplementary Material


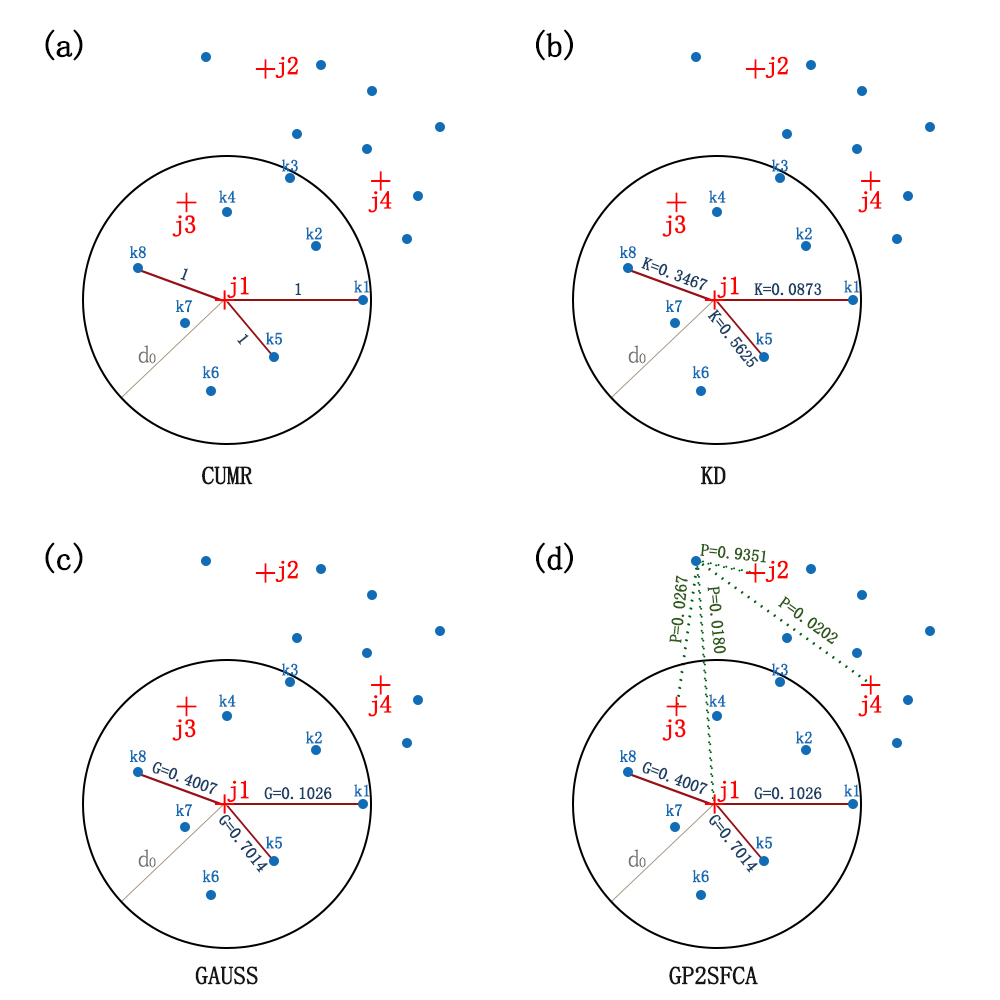


**Supplementary Figure 1.** Diagram of the 2SFCA models. (a) CUMR; (b) KD; (c) GAUSS; (d) GP2SFCA.

**
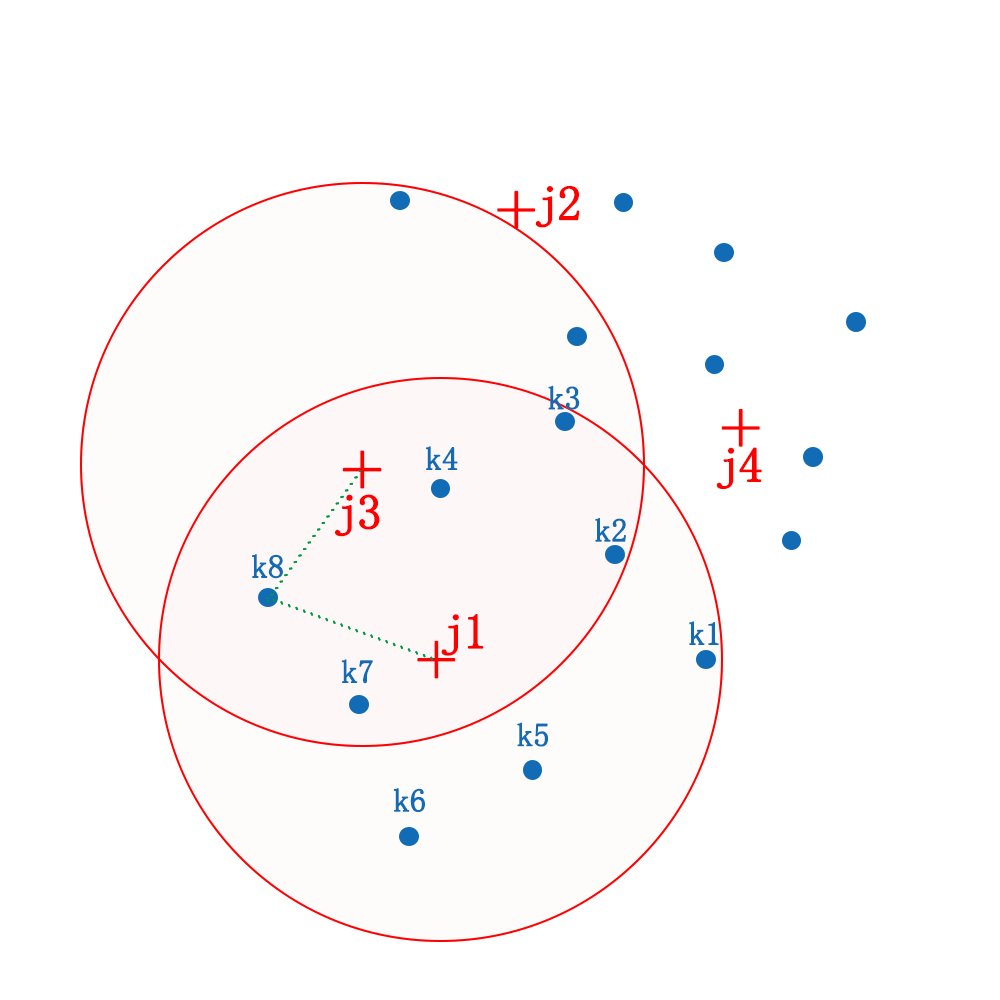
**

**Supplementary Figure 2.** Diagram of the competition of multiple facilities in the catchment area.

**Supplementary Table 1:** Correlation analysis of different distance decay functions

| Model | Correlation Indicator | | Prediction Error Indicators | | | |
| --- | --- | --- | --- | --- | --- | --- |
|  | Pearson correlation Coefficient | P value | MSE | RMSE | MAE | MAPE |
| Cumulative-Opportunity rectangular(CUMR) | 0.0111 | 0.9474 | 294701390 | 17167 | 12582 | 1923.07% |
| Kernel density(KD) | 0.2221 | 0.1803 | 17980567 | 3870 | 2824 | 414.88% |
| Gravity-type Gaussian(GAUSS) | 0.2293 | 0.1661 | 25253886 | 5025 | 3664 | 530.30% |
| GAUSS-Probability(GP) |  |  |  |  |  |  |
| GP0.2 | 0.2768 | 0.0925 | 4749461 | 2179 | 1555 | 79.72% |
| GP0.4 | 0.3551 | 0.0287 | 4432566 | 2105 | 1482 | 76.46% |
| GP0.6 | 0.4500 | 4.59E-03 | 4048068 | 2012 | 1393 | 73.34% |
| GP0.8 | 0.5306 | 6.11E-04 | 3640046 | 1908 | 1301 | 70.89% |
| GP1.0 | 0.5875 | 1.05E-04 | 3261528 | 1806 | 1222 | 70.73% |
| GP1.2 | 0.6278 | 2.45E-05 | 2940927 | 1715 | 1158 | 71.62% |
| GP1.4 | 0.6565 | 7.61E-06 | 2683201 | 1638 | 1110 | 73.34% |
| GP1.6 | 0.6766 | 3.11E-06 | 2480745 | 1575 | 1068 | 74.52% |
| GP1.8 | 0.6901 | 1.64E-06 | 2322498 | 1524 | 1030 | 75.36% |
| GP2.0 | 0.6999 | 1.01E-06 | 2185230 | 1478 | 995 | 75.80% |
| GP2.2 | 0.7026 | 8.76E-07 | 2090059 | 1446 | 968 | 76.67% |
| GP2.4 | 0.7038 | 8.23E-07 | 2013611 | 1419 | 944 | 77.16% |
| GP2.6 | 0.7027 | 8.73E-07 | 1951016 | 1397 | 923 | 77.78% |
| GP2.8 | 0.7022 | 8.96E-07 | 1898607 | 1378 | 906 | 78.25% |
| GP3.0 | 0.6987 | 1.07E-06 | 1860807 | 1364 | 891 | 78.71% |
| GP3.2 | 0.6966 | 1.19E-06 | 1841737 | 1357 | 883 | 79.01% |
| GP3.4 | 0.6933 | 1.40E-06 | 1816380 | 1348 | 875 | 79.55% |
| GP3.6 | 0.6899 | 1.65E-06 | 1795885 | 1340 | 868 | 80.00% |
| GP3.8 | 0.6866 | 1.94E-06 | 1779249 | 1334 | 861 | 80.39% |
| GP4.0 | 0.6835 | 2.25E-06 | 1765689 | 1329 | 857 | 80.93% |
| GP4.2 | 0.6806 | 2.58E-06 | 1754595 | 1325 | 856 | 81.50% |
| GP4.4 | 0.6779 | 2.93E-06 | 1745491 | 1321 | 857 | 82.06% |
| GP4.6 | 0.6753 | 3.30E-06 | 1738009 | 1318 | 858 | 82.56% |
| GP4.8 | 0.6729 | 3.68E-06 | 1731857 | 1316 | 860 | 83.03% |
| GP5.0 | 0.6707 | 4.07E-06 | 1726807 | 1314 | 863 | 83.49% |
